# Emergency Response Resource Allocation in Sparse Network Using Improved Particle Swarm Optimization

**DOI:** 10.3390/ijerph191610295

**Published:** 2022-08-18

**Authors:** Yongqiang Zhang, Zhuang Hu, Min Zhang, Wenting Ba, Ying Wang

**Affiliations:** 1 College of Automobile and Transport Engineering, Nanjing Forestry University, Nanjing 210037, China; 2 Gosuncn Technology Group Co., Ltd., Guangzhou 510663, China; 3 School of Transportation, Southeast University, Nanjing 211189, China

**Keywords:** sparse network, resource allocation, emergency rescue, particle swarm optimization, MATLAB

## Abstract

Western China is a sparsely populated area with dispersed transportation infrastructure, making it challenging to meet people’s accessibility and mobility requirements. Rescue efficiency in sparse networks is severely hampered by the difficulty rescue teams have in getting to the scene of abrupt traffic accidents. This paper develops a layout optimization model for multiple rescue points to address this issue, suggests an improved particle swarm algorithm by including a variation that can reduce optimization time and avoid the disadvantage of precocity, and designs a MATLAB program using an adaptive variation algorithm. The proposed approach increases the effectiveness of rescue in a sparse network and optimizes the distribution of emergency resources.

## 1. Introduction

A sparse network means that road network and other transport facilities are sparsely distributed [1]. It not only limits people’s accessibility and mobility when they are traveling, but also compromises traffic safety, which has troubled both traffic management departments and pertinent study experts [2]. On the one hand, many factors, such as complex and changing road conditions, high speed driving, and fatigued driving are likely to induce emergencies. The proportion of malignant accidents is high. Once accidents occur, they are mostly serious emergencies. On the other hand, due to the sparse population, inadequate emergency detection equipment coverage, a lack of patrol vehicles, and poor communication signals, it is difficult to be found after an accident and to search for the parties involved. In this paper, emergencies are primarily defined as common road traffic accidents, such as significant traffic accidents and significant public health incidents [3,4]. Emergencies have the potential to harm human life and severely destroy property. Emergency management is a focus question that governments pay attention to gradually. In a sparse network, resource allocation is fundamental to emergency management. Effective resource management increases the success of rescue efforts and reduces the loss of life and property. Therefore, in order to improve the effectiveness of emergency responses, resource availability and deployment are crucial [5,6].

In a recent study of emergency response resource allocation [7], we examined three aspects of emergency response: the requirements of emergency incidents and the allocation of response resources; the path from the response resource location to the emergency occurrence; and model-solving algorithms.

Xi et al. developed a hierarchical model of emergency response resource location that combined with a route optimization model. The hierarchical model weighted demand for response services, and the route optimization model consisted of an immune response model coupled with an ant colony algorithm. They made various simplifying assumptions and idealizations, and used locations in Beijing-Tianjin-Hebei region to validate the model and establish its feasibility [8]. Wang et al. analyzed traffic congestion, weather and road conditions to develop a multi-objective model that took account of road network reliability [9]. Caunhye et al. developed a two-part site selection and routing model for emergency response resource allocation and delivery. The authors transformed the two-part model into a single model, which they validated using indicators such as the difference between the expected value and the actual value [10]. Hu et al. considered vehicle type and changes in resources and road network conditions, and used uncertain and dynamic capacity as a dependent variable to characterize the road network [11]. They developed a decision tree to determine resource distribution and used a stepwise hedging algorithm to find the optimum distribution [12,13,14,15].

Recent research into modeling emergency response and resource allocation has made great use of bionic algorithms and particle swarm optimization [16]. However, machine learning algorithms usually converge locally at an early stage of modeling, making it difficult to obtain an optimal global solution to model equations [17,18,19,20].

The allocation of resources for emergency rescue and recovery depends heavily on variables including rescue time, cost, and accident blackspots. Few studies have examined constraints on the type and quantity of rescue resources [21]. Models of resource allocation are difficult to optimize globally and therefore do not have a high success rate in terms of the effectiveness or accuracy of the solutions they provide.

In response to the above problems, this paper makes improvements in two aspects: firstly, it optimizes the research particle swarm algorithm to improve the efficiency of optimal solution solving; secondly, it introduces constraints on the type and quantity of rescue resources based on the consideration of rescue time and cost, the weight of accident black spots and other factors, and establishes an emergency rescue resource allocation model with the objective of minimizing rescue costs.

## 2. Model Description

The goal of modeling is to minimize the rescue cost while ensuring rescue time. Based on this goal, we set up the model below to allocate emergency rescue resources in response resource locations (e.g., service areas, traffic management centers, maintenance centers) available in sparse network according to the blackspot weight of the accident. Emergency rescue resources are including recovery vehicle, tow tractor, crane, fire tender, ambulance and so on.

**Figure 1:**
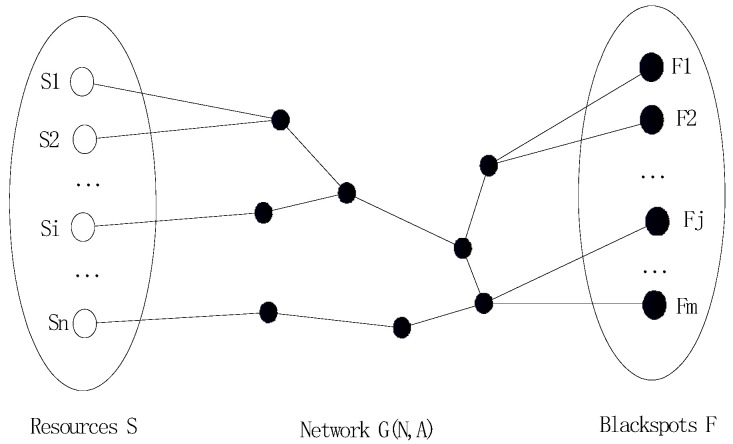
Incident occurrence and allocation of response resources in a sparse network.

We model the geographical area in which an emergency can occur as a sparse network. The nodes represent response resource locations (e.g., service areas, traffic management centers, maintenance depots) and accident blackspots, and the arcs are sections of highways and other roads (Figure 1).

The sparse network is described mathematically as follows. In the network *G*(*N*, *A*), *N* is a set of nodes and *A* is a set of arcs. We define:

(1) The set *S* contains *n* points, and point *S* accommodates resources to a capacity of *a_i_* (*i* = 1, 2, …, *n*);
(2) The set *F* contains *m* blackspots, and each point in *j* requires *F* (*j* = l, 2, …, *m*) response resources;
(3) *λ_ij_* represents the weighting of an available resource from responding point *i* to blackspot *j* in network *G*;
(4) $\omega_{ij}$ represents the weight of blackspot *j* with respect to its serviceability from response point *i*;
(5) *x_ij_* represents the quantity of response resource dispatched from response point *i* to blackspot *j* in network *G*;
(6) *c* is the unit cost of a response resource; *B* is the maximum amount budgeted for response resources.

Blackspot demand for response resources is considered to be an independent variable [22]. Thus, we can define a one-dimensional generalized resource allocation model that considers each response resource as an independent variable [23]. A general stochastic programming model of response resources is shown below that allocates demand probabilities to response points and blackspots:

(1) $$\min\sum_{i=1}^{n}\sum_{j=1}^{m}\omega_{ij}\lambda_{ij}x_{ij}$$

(2) $$\begin{array}{cc} s.t. & \mathrm{Pr}(\sum_{i\in S}x_{ij}\geq r_{j},j\in F)\geq Q,j=1,2,\ldots ,m \end{array}$$

(3) $$x_{i}\leq a_{i},i=1,2,\ldots ,n$$

(4) $$c\sum_{j\in S}x_{i}\leq B$$

(5) $$\sum_{j=1}^{n}x_{ij}=x_{i},i=1,2\ldots ,n$$

(6) $$x_{i},r_{j},a_{i}\in N$$

The objective function Equation (1) minimizes response cost by weighting blackspots. Equations (2)–(6) are the constraints on optimization. Equation (2) represents the constraint that demand is for a limited resource. Equation (3) states that the amount of a resource allocated to a response point is not greater than the quantity of the resource that is available. Equation (4) is the budget constraint, which states that the cost of resources allocated is not greater than the total amount budgeted for that resource, where c is the unit price of a response resource and B is the total budget. Equation (5) defines the conversion of an intermediate variable to a decision-making variable. Equation (6) is the constraint that a decision-making variable must be a nonnegative integer.

## 3. Model Solution

When we have used traditional particle swarm optimization (PSO) to solve complex functions that have several possible local optimal solutions [24,25], traditional PSO has a high probability of failing to escapes from local optimal solutions [26,27], which make us not find the optimal solution.

So we have based on the concept of mutation used in the genetic algorithm and optimized the PSO by introducing the inertia weight coefficient (a mutation operation), in order to expands the population search space, which otherwise continually contracts across iterations, thus enabling particles initialize the their position and velocity in model with a given probability to jump out of the highest quality position previously searched and carry out searches in a larger space, where the optimized PSO wound maintains population diversity and improve the probability in finding the global optimal solution, especially in solving complex functions [17,18,19,20,21,22]. Therefore, we optimize PSO to expand the population search space by introducing the inertia weight coefficient based on the concept of mutation in the genetic algorithm, which enables particles initialize their position and velocity in model with a given probability to jump out of the highest quality position previously searched and carry out searches in a larger space. The optimized PSO would maintain population diversity and improve the probability in finding the global optimal solution, especially in solving complex functions [28,29,30,31].

The inertia weight is adjusted in Equations (7) and (8), and a constriction factor is introduced [32,33,34,35]. All particles in PSO iteratively update their speed and location according to Equations (7) and (8).

(7) $$v_{id}^{\left(t+1\right)}=w*v_{id}+c_{1}*r_{1}*\left(pb_{id}^{\left(t\right)}-x_{id}^{\left(t\right)}\right)+c_{2}*r_{2}*\left(gb_{id}^{\left(t\right)}-x_{id}^{\left(t\right)}\right)$$

(8) $$x_{id}^{\left(t+1\right)}=x_{id}^{t}+v_{id}^{\left(t+1\right)}$$

where: 

*w* is the inertia weight coefficient; *i* = 1, 2, …, *m* and *m* is the total number of particles; 

*d* = 1, 2, …, *n* and *n* is the dimension of the target search space;

*t* is the current evolutionary generation;

$v_{id}^{\left(t\right)}$ between $-v_{max}$ and $v_{max}$ refers to the velocity’s *d* vector of particle *i* in the *t* generation searching optimization;

$x_{id}^{\left(t\right)}$ refers to the location’s d vector of particle *i* in the *t* generation searching optimization;

$pb_{id}^{\left(t\right)}$ refers to the best location’s *d* vector of particle *i* in the *t* generation searching optimization;

$gb_{id}^{\left(t\right)}$ refers to the best location’s *d* vector of group particles in the *t* generation searching optimization;

*r*_1_ and *r*_2_ are random numbers in the interval [0, 1]; 

*c*_1_ and *c*_2_ are learning factors which are used to adjust the largest step length of a particle searching optimization. 

**Figure 2:**
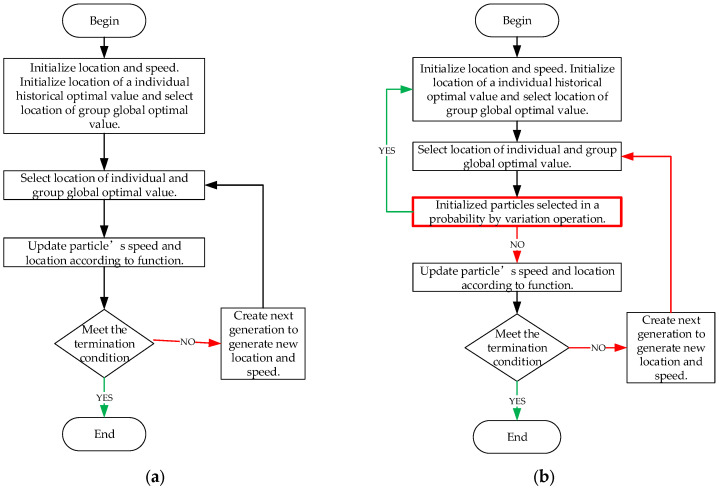
Two target PSO algorithm flowcharts for comparison: (**a**) standard PSO algorithm; and (**b**) optimized PSO algorithm. The red text box shows the mutation operation introduced in this study.

Two target PSO algorithm flow charts are shown for comparison in Figure 2. In compare with the standard PSO, the optimized PSO has a remarkable difference where adds a step: initialized particles could be selected in a given probability and restart search the potential optimization.

**Figure 3:**
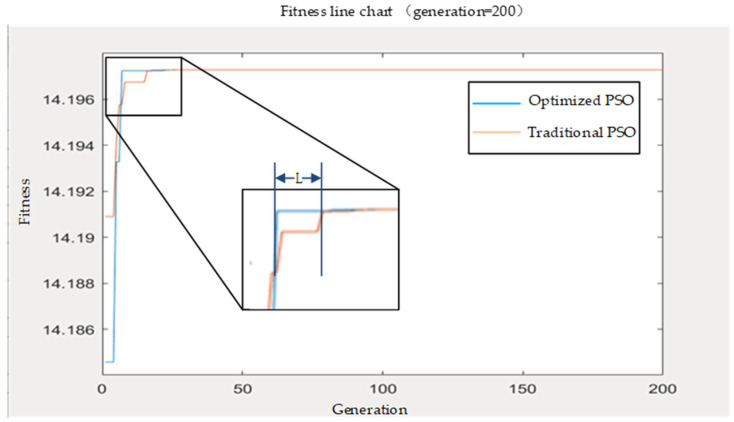
Comparison of optimized processes using MATLAB.

MATLAB was used to simulate and compare two algorithms including standard PSO and optimized PSO. The learning factors *c*_1_ and *c*_2_ are both given the value 1, the inertia weight is 0.8, the maximum number of iterations is 200, and the number of individuals in the particle swarm *N* is 20. The resulting optimal individual fitness values are shown in Figure 3. It can be seen from the results that the particle swarm algorithm of the mutation operator can jump out of the local minimum point to get better results.

Figure 3 shows that the optimized algorithm has a fitness value approaching 0 and is almost certainly convergent before 20 generations. In the inset, L along the x-axis indicates that optimized PSO requires fewer evolutionary iterations than standard PSO to reach 0 fitness. We found that the advantages in Figure 3 are as follows: (1) the optimization algorithm is able to iterate for fewer generations for the same confidence value. (2) the optimization algorithm can obtain better confidence values for the same number of iterations. Thus, optimized PSO is better than standard PSO in global optimization and rate of convergence. The advantage of the optimization algorithm is the ability to obtain the global optimal solution with a higher probability.

## 4. Model Validation

### 4.1. General Situation

**Figure 4:**
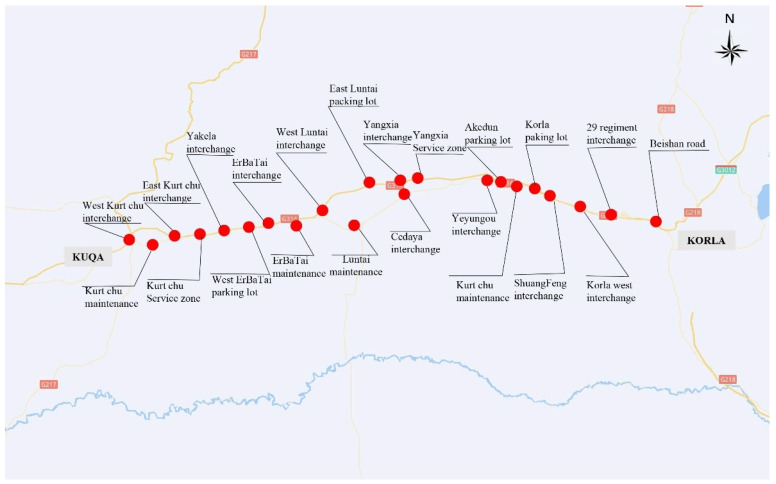
Regional sparse network in Aksu-Korla.

A section of the sparse network between Aksu (in the west) and Korla (in the east) from Kuqa to Korla, referred to as KK, in Xinjiang (Figure 4) was used to validate the model [36,37,38]. The resources considered for allocation were recovery vehicles, fire tenders and ambulances.

**Figure 5:**
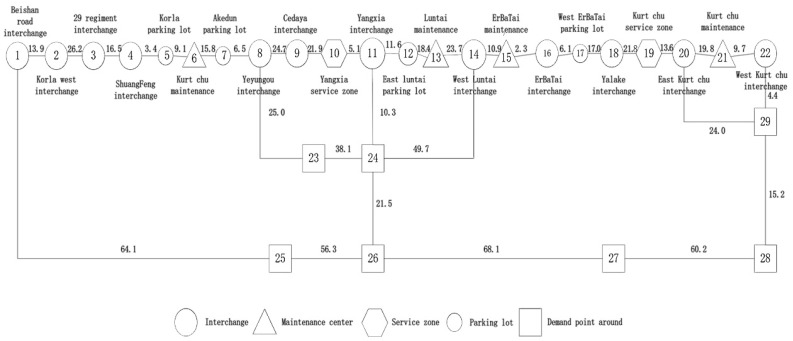
Regional sparse network KK.

We abstracted the road network (Figure 5) from the map (Figure 4) by extracting the interchanges, parking lots, service areas and maintenance depots in the road as network nodes.

In Figure 5, numbers on link represent distances between nodes (km) according to traffic survey. The nodes from Beishan road interchange to the western interchange of Kuqa are numbered 1–22; the demand nodes and emergency response nodes which are not on highways are numbered 23–29. Existing response points at maintenance depots are Kurt chu (6), Luntai (13), ErBa Tai (15) and Kuqa (21). There are four maintenance depots and two service areas. We set *n* to 4 for recovery vehicle allocation and to 2 for allocation of emergency vehicles.

### 4.2. Setting Model Parameters

The parameter to be optimized is the decision variable *x_i_*; *x_ij_* is an intermediate variable; parameters to be set are *ω_j_*, *λ_ij_*, *Q*, *r_j_* and *a_j_*.

1. Blackspot weight *ω_j_*

We calculated the blackspot weights based on incident level and probability of occurrence. The weights of the blackspot are shown in Table 1.

2. Time weight *λ_ij_* from response point to blackspot

We chose response travel time as a weight. We assumed that the average speed of a recovery vehicle is 60 km/h, the average speed of an ambulance is 100 km/h, the average transition time driving from a highway via an offramp to the ordinary sparse network (or in the opposite direction) is 4 min, and that the average time to drive through the barrier to turn around is 2 min. An emergency response vehicle is not impeded by a construction zone or not bound to overtake vehicles on the ordinary sparse network, and we assumed barriers could turn around anywhere in the network.

Maintenance depots were numbered 1–4, service areas 1–2. A blackspot was defined as a highway location at which there had been at least 30 accidents, according to historical regional incident data. The speed of a recovery vehicle is unchanged if it is driven in the area of an accident, so we did not consider the effect of the incident on travel time. The speed of a fire tender is 100 km/h when the vehicle is not affected by the incident. On roads around an accident, vehicle speed decreases due to traffic tailback or lack of access to the scene of the accident. We do not consider the effects of traffic queues caused by an accident on fire and rescue vehicles as they are difficult to quantify and predict. The time weights from maintenance depots and service areas to blackspots are shown in Table 2.

3. Response service levels *Q*

Response service levels are as follows. *Q* = 1 indicates that response points can meet the needs of all blackspots simultaneously, which is impractical. *Q* = 0.9 is too high which leads to waste of resources. In general, *Q* should be 0.7 or 0.8. We used these two values for comparison.

4. Quantity of response resource *r_j_*

The random variable *r_j_* has a Gaussian distribution approximately. Response resources for different blackspots have Gaussian distributions of different means and variances. The desired value of a response resource needed at different blackspots can be obtained from historical accident and response data.

We assumed that all response resources have Gaussian distributions with variance 1. The response resources (small, medium and large recovery vehicles, tow tractors, cranes, fire tenders and ambulances) for 8 different blackspots have the Gaussian distributions shown in Table 3.

5. Capacity of response points *a_j_*

The capacity of a response point may be its planned or actual capacity. There are six response points (*a_j_* = 6).

### 4.3. Allocation Result and Analysis of Self-Adaption PSO

**Figure 6:**
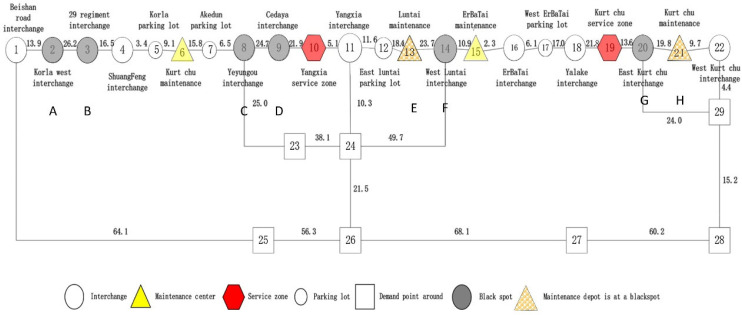
Regional road network showing resource allocation.

At a service level of 0.7, taking the response vehicles to be allocated as an example, we obtained optimal allocation of response vehicles at every response location by using PSO, as shown in Table 4 and Figure 6.

Table 4 and Figure 6 show that maintenance depot 13 is allocated more resources than other maintenance depots, but the differences are not great.

We performed an analysis with the weights shown in Table 2, using the small recovery vehicle as an example. Maintenance depot 13 is close to blackspots C, D, E and F all whose weight is less, so it is reasonable to allocate more response resources to maintenance depot 13.

We analyzed the demand for resources. Demand is greater from blackspots A and C, so it is also reasonable to allocate more response resources to maintenance depot 13.

We analyzed the blackspot weights. The weights of blackspot C, D and E are 0.375, 0.25 and 0.375; the least (D) should be considered first because of the objective function. Thus it is reasonable to allocate more response resources to maintenance depot 13.

Figure 6 show that more fire tenders and ambulances are allocated to service area 1 than to service area 2. Service area 1 is close to blackspots A, B, C and D, which have accident levels of 2, 4, 1 and 3, so it requires more response resources. Thus, it is reasonable to allocate more fire trucks and ambulances to service area 1.

### 4.4. Summary

The analysis of the examples validated the model and showed that it is feasible for practical use. In solving the resource allocation problem, as the resources required for the accident at a blackspot are random, the random variables generated in each calculation of the particle swarm algorithm will be different, resulting in small differences in the results, which is in accordance with the situation found in practice.

## 5. Conclusions

The objective was to minimize the cost of responding to accidents at blackspots, constrained by resource availability. We created a multivariate emergency response resource allocation model. By introducing the inertia weight coefficient, the position and velocity of model particles are initialized with a given probability and the optimization algorithm is iteratively solved. The model and algorithm were validated using MATLAB. The main conclusions are as follows:

1. A budget-constrained resource allocation model can determine minimum resource allocation for rescue costs, given the capacity of the resource locations;
2. The improved algorithm is simple and easy to implement. The algorithm only needs to determine how to represent the particles as problem solutions and iteratively find the optimal solution by guidance of the local optimal solution and the global optimal solution. There are no operations such as crossover. The principle is simple with few parameters and simple coding;
3. When the objective function is extremely complex, especially extreme points are multiple, the traditional algorithm is prone to fall into local optimal solutions. However, the improved algorithm has a strong global search capability to find the optimal value. It has a greater advantage in solving models with extremely complex objective functions;
4. For complex systems, the iteration time is longer due to the introduction of adaptive variation and a larger number of samples for stochastic simulations.

The results of this paper will assist emergency managers in creating effective emergency rescue plans in sparse network. It provides a strong theoretical basis for emergency decision-making.

Further research into the following two aspects of this study is planned. Ramps, interchanges, toll stations and other facilities will be added to create a more comprehensive and detailed sparse network abstract map; and further research into emergency management of the urban road network will be undertaken.

## Figures and Tables

**Table 1:** Blackspot weights.

Blackspots	A	B	C	D	E	F	G	H
Incident level	2.000	4.000	1.000	3.000	3.000	4.000	3.000	4.000
Probability	0.500	0.750	0.250	1.000	0.750	1.000	1.000	0.500
Weight	6.000	9.000	6.000	4.000	6.000	6.000	4.000	16.000
Uniform weight	0.375	0.5625	0.375	0.25	0.375	0.375	0.250	1.000

**Table 2:** Time weight from maintenance depots and service area to blackspots (average running time, min).

**Maintenance Depots**								
**Number**	**A**	**B**	**C**	**D**	**E**	**F**	**G**	**H**
1	21	32	35	72	63	86	112	89
2	36	14	31	48	46	60	86	128
3	31	46	15	22	12	38	64	41
4	86	92	78	68	59	24	8	40
**Service Area**								
**Number**	**A**	**B**	**C**	**D**	**E**	**F**	**G**	**H**
1	0.1	31.6	10.8	34.2	23	44.6	59.6	40.4
2	31	33.2	21	4	21.2	27.2	42	39.2

**Table 3:** Gaussian distribution of resource and response vehicles from 8 blackspots.

Blackspots	A	B	C	D	E	F	G	H
Small recovery vehicle	N (2, 1)	N (1, 1)	N (2, 1)	N (1, 1)	N (1, 1)	N (1, 1)	N (1, 1)	N (1, 1)
Medium recovery vehicle	N (1, 1)	N (0, 1)	N (1, 1)	N (1, 1)	N (1, 1)	N (0, 1)	N (1, 1)	N (0, 1)
Large recovery vehicle	N (0, 1)	N (0, 1)	N (1, 1)	N (0, 1)	N (0, 1)	N (0, 1)	N (0, 1)	N (0, 1)
Tow tractor	N (1, 1)	N (0, 1)	N (1, 1)	N (0, 1)	N (0, 1)	N (0, 1)	N (0, 1)	N (0, 1)
Crane	N (1, 1)	N (0, 1)	N (1, 1)	N (0, 1)	N (0, 1)	N (0, 1)	N (0, 1)	N (0, 1)
Fire tuck	N (1, 1)	N (0, 1)	N (1, 1)	N (0, 1)	N (0, 1)	N (0, 1)	N (0, 1)	N (0, 1)
Ambulance	N (1, 1)	N (0, 1)	N (2, 1)	N (1, 1)	N (1, 1)	N (0, 1)	N (1, 1)	N (0, 1)

**Table 4:** Allocation result of every rescue vehicle (*Q* = 0.7).

Response Locations	Recovery Vehicle			Tow Tractor	Crane	Fire Tender	Ambulance
	Small	Medium	Large				
Maintenance depot 6	2	2	1	2	2	-	-
Maintenance depot 13	4	4	2	1	2	-	-
Maintenance depot 15	3	2	1	1	1	-	-
Maintenance depot 21	2	2	1	2	1	-	-
Service area 1	-	-	-	-	-	2	2
Service area 1	-	-	-	-	-	1	1

## Data Availability

Not applicable.
